# The co-word analysis and visualization of a 10-year international research on drowning

**Published:** 2019-07

**Authors:** Majid Pourshaikhian, Sadegh Moshtaghi Koojel, Fatemeh Shafaee, Aboozar Ramezani

**Affiliations:** ^*a*^Department of Emergency and Disaster Health, School of Paramedical, Guilan University of Medical Sciences, Rasht, Iran.; ^*b*^Research Committee, School of Nursing, Midwifery and Paramedical, Guilan University of Medical Sciences, Rasht, Iran.; ^*c*^Department of Nursing Anesthesiology, School of Nursing, Midwifery and Paramedical, Guilan University of Medical Sciences, Rasht, Iran.; ^*d*^Department of Medical Library and Information Sciences, Tehran University of Medical Sciences, Tehran, Iran.

## Abstract

**Background:**

WHO statistics shows that drowning is one of the most important research areas in the world. Despite the extensive research on drowning in the past, scientometric approaches have not been used yet to evaluate these studies.

**Methods:**

In this descriptive cross-sectional study, which is based on scientometric method, the analysis was done using the words in the title, abstract and keywords. In addition to visualization of the universal cooperation between universities and researchers, the bibliographic and citation data of articles have been retrieved from Web of science, PubMed and Scopus in the period of 2007-2017. These documents were analyzed using HistCite12, 03, 1 and VOS viewer 6, 5, 1 software.

**Results:**

2314 articles in Web of science, 4024 articles in Scopus and 98 articles in PubMed published in the past 10 years were found. The number of articles published by Iranian researchers in Web of Science, Scopus and PubMed were 19, 45 and 10, respectively. The findings showed that the number of articles about this topic had increased in Web of Science and Scopus over the last 10 years. According to the results of this study, important research areas were visualized in the three databases by extracting repeated words. The frequency pattern of words showed that the focus of the research has been on cardiovascular diseases, age-groups, resuscitation problems, forensic medicine, and suicide, among others. The language of more than 80% of the documents was English. American and English Universities, journals and researchers had the most contribution in this field.

**Conclusions:**

The result of this study will help researchers to develop, plan and increase the qualitative and quantitative levels of their research on drowning. Similar to developed countries, developing research can help improve the culture and general education to prevent drowning in developing countries.

**Keywords:**

Drowning, Scientometrics, Scientific Productions, Article

